# Osteosarcoma of the larynx: a case report

**DOI:** 10.1186/1757-1626-1-365

**Published:** 2008-12-01

**Authors:** Giampiero Mottola, Anna Maria Cascone, Matteo Cavaliere, Basilio Angrisani, Gabriella Fiorillo, Giuseppe Parente, Fabrizio Volino, Maurizio Iemma

**Affiliations:** 1 Department of Otolaryngology, Hospital "San Giovanni di Dio e Ruggi d'Aragona", Via San Leonardo, Salerno, Italy; 2 Department of pathology, Hospital "San Giovanni di Dio e Ruggi d'Aragona", Via San Leonardo, Salerno, Italy; 3 Department of pathology, Hospital "Gemelli", Rome, Italy; 4 Department of Nuclear Medicine, 2nd University of The Study of Naples, Italy

## Abstract

**Background:**

We add a new rare illustrative case of osteosarcoma of the larynx to the literature.

**Case presentation:**

The patient (man; 56 years old) first underwent several biopsies, followed in the end by a total laryngectomy. Diagnosis was histological. The patient developed regional and distant metastases and died of disease after 3 months from surgery.

**Conclusion:**

Osteosarcoma of the larynx is a rare and aggressive tumour with a poor long-term prognosis. The preferred treatment for this tumour is aggressive surgery.

To perform a diagnosis we must be in presence of osteoid or of neoplastic osseous tissue directly produced by the neoplastic cells.

## Background

Osteosarcoma is a rare larynx tumor [1-9]. From 1989 to 2008 our pathology and histology department reported only one case. Mesenchymal tumours add up to only 0,32 – 2% of all laryngeal neoplasms, and among them osteosarcoma is the rarest. We add a new illustrative case to the 15 cases of primitive tumours reported in literature [1-9].

## Case presenation

A 56 years old Caucasian man, butcher, weight 176 lbs, Height 6', came to our department after he had been suffering from acute dyspnea, dysphagia and dysphonia for 4 months. He did not smoke nor did he use to drink alcohol. He had hypertension, some months before he had undergone an angioplasty and a substitution of a cardiac valve.

A physical examination performed by fiberoptic laryngoscopy revealed a diffused larynx oedema with reduction of glottic space and vocal cord mobility. No palpable laterocervical lymph nodes were observed. The first procedure to be performed was a tracheostomy to reduce the stridor. At the same time some biopsies were performed via direct laryngoscopy. The first biopsy revealed a mesenchymal neoplasia with spindle cells, showing slight atypias and low mitotic rate and which were negative to immuno-istochemical staining with S100, actin, and CK, with focal positivity on p53 and immersed in a myxoid stroma.

**Figure 1:**
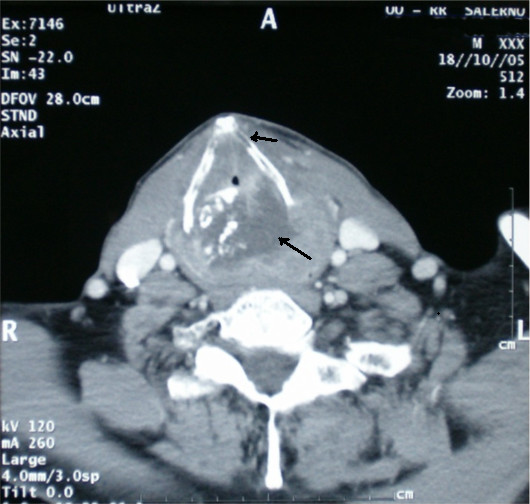
CT Scan with contrast. Tumour mass affecting glottic and supraglottic larynx, which involved the thyroid cartilage and the paralaryngeal space. In the context of the mass presence of calcification areas.

Tumuor staging by means of total body CT scanning with contrast revealed a tumour mass affecting glottic and supraglottic larynx, which also affected the thyroid and circoid cartilages and extended to the paralaryngeal space (Fig. 1). No regional or distant metastases were identified. A new laryngoscopy was performed. The new biopsies confirmed the previous examintions.

**Figure 2:**
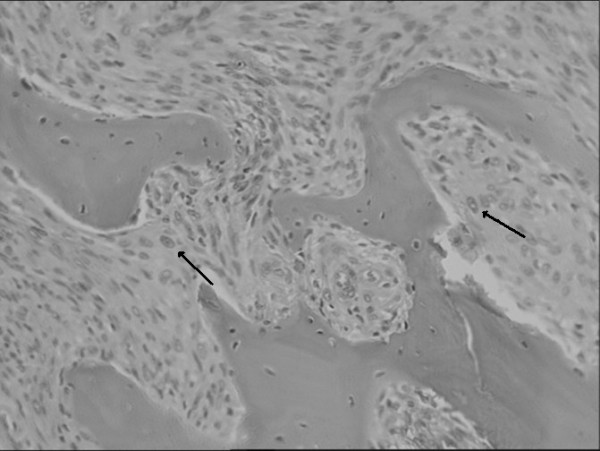
Atypical spindle tumor cells grow between irregularly bone trabeculae and osteoid tissue (Ematoxilin-eosin, 10×).

Finally we got an informed consent for a total laryngectomy. Intraoperative frozen sections revealed a malignant mesenchymal neoplasm. The final diagnosis based on the permanent sections was osteosarcoma of the larynx (Fig. 2).

We administer adjuvant therapy (Isofosfamide 3 mg/m^2 ^for 3 days i.v. – Farmorubicina 50 mg/m^2 ^for a day) but the patient developed distant metastases to the lungs and died of disease after 3 months.

## Discussion

Osteosarcoma of the larynx is a very rare and aggressive tumor because of hematogenous spreading, and as it is difficult to be diagnosed [3]. Only few cases of primary larynx osteosarcomas are described in literature, and all of them have a poor prognosis. From 1942 [1-9] to 2008 only fifteen cases were reported (M/F ratio 13:1; average age: 65). Of the fiftheen cases reported in literature, twelve were treated with surgery, two received a primary radiation therapy, and one had been treated with radiotherapy three years before for a squamous cell carcinoma of the larynx, and we do not know which therapy he was treated with [2,3]. Three of the five patients treated with radiation therapy died of disease, and no results are available about chemotherapy, as only a case received this treatment, and presented local recurrence. Laterocervical lymph node metastases are a late event.

Local recurrence occurred after an average time of 9.5 months [2,3]. Average time of distant spreading was 11.4 months and about 50% of the cases died of disease from 3 to 20 months after surgery [2,3].

We administer adjuvant therapy also if literature data are controversial and not reliable [2,3,7]. In our research we found some negative and misleading biopsies. On fiftheen cases described in the literature, before the surgery four were classified as lesions different from osteosarcoma [3].

The diagnosis of this tumour is very difficult because of the involvement of the submucosa. This is the reason why several biopsies performed via microlaryngoscopy are unreliable to stage the lesion.

After total laryngectomy the back face of the larynx appears macrosopically evenly deformed by the presence of the tumor mass, superficially covered by pink mucosa, solid to cut, of hard-elastic consistency with calcific areas, of white-yellowish colour and with brownish foci.

Histologically the tumour appears to be mostly build of spindle cells, which show the setting of bundles. The cells show plump, hyperchromatic and atypical nuclei with an increase in the mitotic index. A well differentiated chondroblastic neoplastic component is also present, with slight cytologic atypias represented by areas with binucleated cartilaginous cells, with many cells per laguna, and with focal areas of ossification and deposition of osteoid matter. The presence of osteoid tissue and neoplastic osteoid tissue, not connected with cartilaginous tissue, made diagnosis possible (Fig. 2).

## Conclusion

Osteosarcoma of the larynx is a rare aggressive tumor that affects predominantly men [1-9]. Anamnesis is negative for smoke or alcohol history. Tipical presentation is with dyspnea, dysphonia, dysphagia and hoarsness, benign appearing at fiberoptic laryngoscopy without mucosal infiltration, no palpable neck masses. CT scan shows a destructive lesion of the larynx often within calcification areas. Demolitive surgery is the treatment of choice while radiation or chemotherapy seems not to improve the prognosis. We think that deep biopsies could make diagnosis more reliable, but not certain. Only after a histological examination of the entire larynx did we get to the final diagnosis of osteosarcoma: osteoid or neoplastic osseous tissue directly produced by the neoplastic cells.

## Competing interests

The authors declare that they have no competing interests.

## Consent

Written informed consent was obtained from the patient for publication of this case report and accompanying images. A copy of the written consent is available for review by the Editor-in-Chief of this journal.

## Authors' contributions

GM and MC analyzed and interpreted patient's data; AMC, BA and GF performed the histological examination; GP, FV got literature dates; MI performed surgery. All authors read and approved the final manuscript.

**Figure 3:**
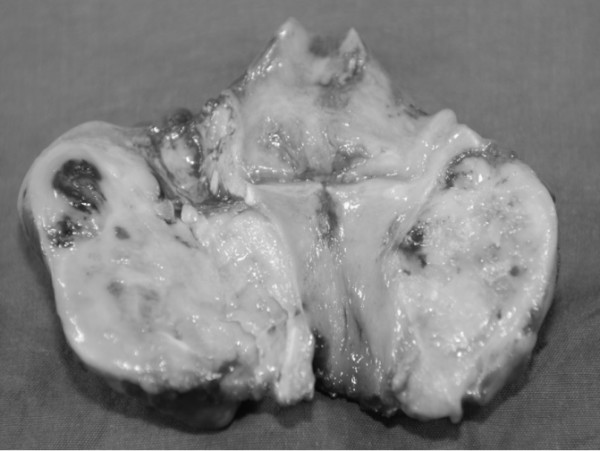
Postoperative aspect of the tumor.

## References

[B1] Arellano B, Gonzalez FM, Martinez G, Salas C, Ramirez Camacho R, Vergara J, Vicente J, Vargas JA (1999). Inflammatory pseudotumor of the larynx. Acta Otorrinolaringol Esp.

[B2] Athre RS, Vories A, Mudrovich S, Ducic Y (2005). Osteosarcomas of the larynx. Laryngoscope.

[B3] Berge JK, Kapadia SB, Myers EN (1998). Osteosarcoma of the larynx. Arch Otolaryngol Head Neck Surg.

[B4] Dahm LJ, Schaefer SD, Carder HM, Vellios F (1978). Osteosarcoma of the soft tissue of the larynx: Report of a case with light and electron microscopic studies. Cancer.

[B5] Kassir RR, Rassekh CH, Kinsella JB, Segas J, Carrau RL, Hokanson JA (1997). Osteosarcoma of the Head and neck: Meta-analysis of nonrandomized studies. Laryngoscope.

[B6] Madrigal FM, Godoy LM, Daboin KP, Casiraghi O, Garcia AM, Luna MA (2002). Laryngeal osteosarcoma: a clinicopathologic analysis of four cases and comparison with a carcinosarcoma. Ann Diagn Pathol.

[B7] Rossi RM, Landas SK, Kelly DR, Marsh WL (1998). Osteosarcoma of the larynx. Otolaryngol Head Neck Surg.

[B8] Topaloglu I, Isiksacan V, Ulusoy S, Sisman S (2004). Osteosarcoma of the larynx. Otolaryngol Head Neck Surg.

[B9] Williams SB, Foss RD, Ellis GL (1992). Inflammatory Pseudotumors of the Major Salivary Glands. Clinicopathologic and immunohistochemical analysis of six cases. Am J Surg Pathol.

